# Diagnosis and Treatment of a Recurrent Bleeding Dieulafoy’s Lesion: A Case Report

**DOI:** 10.7759/cureus.32051

**Published:** 2022-11-30

**Authors:** Amanda R Levy, Sierra Broad, James R Loomis III, Julie A Thomas

**Affiliations:** 1 Family Medicine, New York Institute of Technology College of Osteopathic Medicine, Glen Head, USA; 2 General Surgery, Lake Erie College of Osteopathic Medicine, Erie, USA; 3 Family Medicine, Sisters of Charity Hospital, Buffalo, USA

**Keywords:** catheter angiogram, esophagogastroduodenoscopy (egd), recurrent gi bleeding, rare case report, case report, antiplatelet, hematemesis, upper gastrointestinal bleed, dieulafoy’s lesion

## Abstract

Dieulafoy's lesions are uncommon causes of upper gastrointestinal bleeding (UGIB) that pose a life-threatening risk if not diagnosed promptly and treated appropriately. These lesions are composed of enlarged submucosal blood vessels that bleed despite any gross abnormality. Early intervention with esophagogastroduodenoscopy (EGD) is necessary to avoid more invasive treatment with angiogram embolization or surgical removal. This paper aims to discuss a case regarding a patient with difficult-to-control recurrent bleeding from a Dieulafoy's lesion located in the gastric fundus of a previously healthy 60-year-old female. This case highlights the need for dual therapy and special considerations regarding antiplatelet medications and supplements when treating patients with Dieulafoy's lesions.

## Introduction

Upper gastrointestinal bleeding (UGIB) is a common health problem, with patients typically presenting with symptoms such as hematemesis, coffee-ground emesis, or melena. It is estimated that the annual incidence of UGIB is approximately 80 to 150 per 100,000 population [1]. The most common cause of UGIB is typically attributed to peptic ulcer disease; however, other rare causes of UGIB should be included in the differential, given the serious nature of this disease process and the risk of life-threatening bleeding [1]. Dieulafoy's lesions are rare vascular malformations of the gastrointestinal (GI) tract that account for approximately 1% to 2% of acute GI bleeding due to developmental vascular malformations in the GI tract. These lesions represent enlarged submucosal blood vessels that bleed without any overlying abnormality. Often, these lesions arise along the lesser curvature of the stomach within 6 centimeters (cm) of the gastroesophageal junction, a region typically supplied by the left gastric artery [2]. Prompt recognition and diagnosis of these lesions are imperative because these lesions tend to cause severe, life-threatening, and recurrent GI bleeding. Endoscopy serves as the first diagnostic test of choice; however, it only has a 70% diagnostic yield because these lesions are typically small and difficult to identify [3]. Despite this difficulty, a decrease in the mortality rate, of 80% to 8.6%, has been shown due to advancements in endoscopy [4]. An extensive literature review has shown a lack of consensus on prompt diagnosis and treatment of these lesions; however, therapeutic endoscopy can control bleeding in up to 90% of patients [4]. Endoscopic monotherapy results in higher rates of re-bleeding; therefore, therapeutic endoscopy, including dual therapy of hemoclip placement and hemostasis with a sclerosis agent, is considered the first step in the diagnosis and management of Dieulafoy's lesions [4-5]. Often, a history of the present illness involves a sudden onset of massive and recurrent hematemesis, which leads to difficulty locating the site of the lesion during endoscopy [6]. The purpose of this case study is to further delineate the importance of early recognition and prompt treatment of Dieulafoy's lesions as a cause of UGIB, given the life-threatening nature of this condition.

## Case presentation

A previously healthy, 60-year-old Caucasian female with a past medical history of anxiety, obsessive-compulsive disorder, and depression presented to the emergency department (ED) due to intermittent abdominal pain for approximately five days followed by black tarry stools, weakness, and shortness of breath. Complete blood count (CBC) indicated a decrease in hemoglobin (Hgb) to 10.6 g/dL from 14.5 g/dL three months prior with positive stool guaiac. She denied any recent non-steroidal anti-inflammatory drug (NSAID) use, alcohol use, drug use, or smoking history and any past history of GI bleeds. Surgical history included ovarian cyst removal. Home medications included sertraline tablet 150 mg by mouth daily, Vitamin E, and Vitamin D.

Shortly after admission for a GI workup, the patient experienced hematemesis of 200 milliliters (mL) of bright red blood (BRB) and dark red clots, hypotension of 99/61, and tachycardia of 160 beats per minute (bpm). A rapid response alert was called, leading to emergency intubation with mechanical ventilation sedated on propofol, transfusion of three units of packed red blood cells, and interventional radiology (IR) consultation for a mesenteric angiogram. No active bleed was noted by IR; therefore, no intervention was performed. She was then transferred to the intensive care unit (ICU).

**Figure 1:**
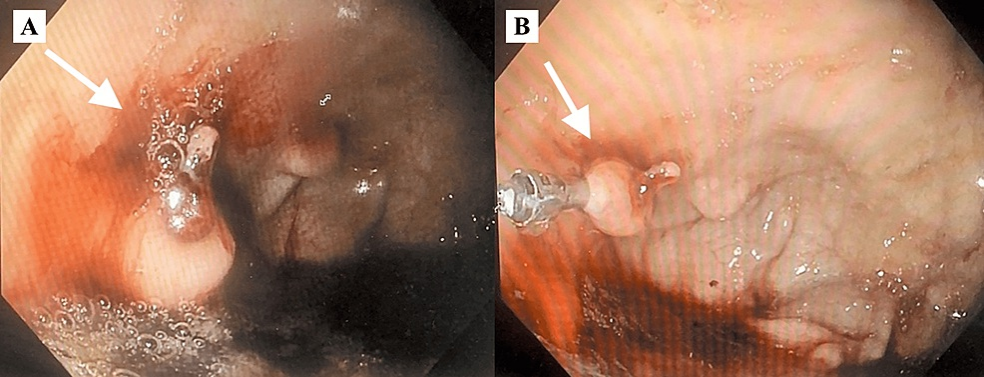
Initial EGD 1A: 1.0 cm protuberance; 1B: 0.5 cm protuberance EGD: esophagogastroduodenoscopy

Esophagogastroduodenoscopy (EGD) was performed (Figure 1), which showed a 1.0 centimeter (cm) protuberance with a submucosal impression at the gastric fundus with a visible large bleeding arterial vessel at the site of the protuberance (Figure 1A). One endoclip was placed at this site. An additional 0.5 cm protuberance with overlying normal mucosa was found adjacent to the previously described protuberance (Figure 1B). Limitations to the EGD included the amounts of fresh blood with clots noted in the gastric body, antrum, and fundus in addition to the duodenum.

The patient was extubated and transferred to the floor once medically stable. At this point, the patient was started on pantoprazole 40 mg by mouth twice per day. Her Hgb continued to decrease despite packed red blood cells (PRBC) transfusion from 8.6 g/dL to 7.4 g/dL. After being stable for seven hours, a second rapid response was initiated due to the patient experiencing hematemesis of 200 mL BRB. Her stool was dark and the fecal occult blood test was heme occult positive. She was promptly transferred to the ICU.

A second EGD was performed with improved visibility of the lesion due to decreased clots at the site. This indicated the presence of a Dieulafoy's lesion at the previously noted 1.0 cm protuberance location. An additional three clips were deployed at the vessel, resulting in a complete cessation of bleeding. The patient was discharged, as she was now hemodynamically stable.

**Figure 2:**
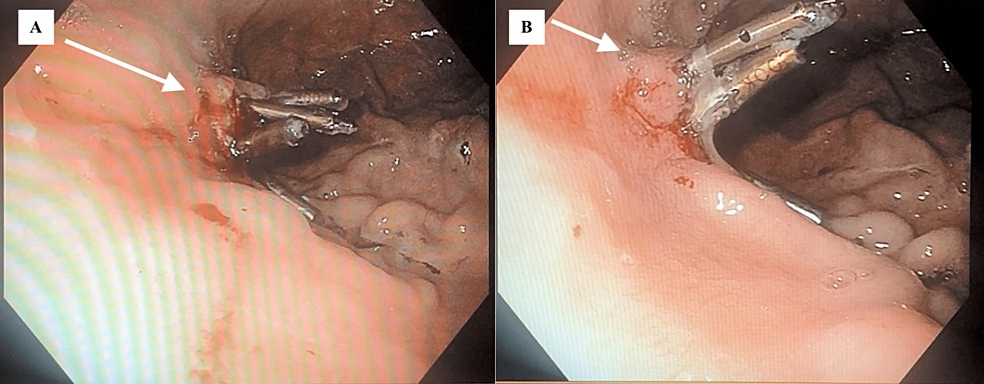
Third EGD 2A: Pre-gold probe cautery; 2B: Post-gold probe cautery EGD: esophagogastroduodenoscopy

She remained stable until approximately seven days post-discharge when she experienced a near syncopal event and returned to the ED. Shortly after arrival, the patient experienced multiple episodes of coffee ground emesis and one dark, tarry stool with decreasing Hgb from 10.4 g/dL immediately post-discharge to 6.4 g/dL and a decrease in platelets (PLTs) from 211,000/mm^3^ to 136,000/mm^3^. She was admitted to the ICU for acute hemorrhagic shock secondary to a massive UGIB and received 4 units of PRBC, two fresh frozen plasma (FFP), and an emergent EGD (Figure 2). The emergent EGD demonstrated the previously placed clips were covered in the coffee ground residue (Figure 2A). Extensive lavage and suctioning were performed at the site of the lesion, which led to scant oozing from the clipped site. Gold probe cautery to the clips was used with epinephrine injected into the lesion to stop continued bleeding (Figure 2B). No bleeding was noted after the procedure for approximately one day before hematemesis recurred. IR performed a mesenteric angiogram. No evident bleeding was noted during the angiogram’s duration, so the gastroduodenal artery was embolized at this time. The patient remained stable and was transferred from the ICU to the medical floor.

After approximately 96 hours of observation, she experienced another episode of hematemesis involving 400 mL of BRB. An emergent surgical consult was ordered; however, they felt the patient needed a repeat mesenteric angiogram with embolization of a different arterial source. IR then embolized the right gastroepiploic artery. The patient was observed for an additional 96 hours for observation of diet toleration and trending CBC. PLTs and Hgb trended upward from 93,000 /mm^3^ to 134,000 /mm^3^ and 8.3 g/dL to 10 g/dL, respectively. No further hematemesis occurred.

Her sertraline was decreased from 150 mg to 100 mg with the plan to slowly taper off the medication, and she was advised that vitamin E has antiplatelet effects and to not overuse this supplement. Given that the patient was hemodynamically stable, she was discharged to sub-acute rehab (SAR) with anticipation of outpatient follow-up and CBC within one week of SAR discharge.

## Discussion

Dieulafoy's lesions

Dieulafoy’s lesions continue to be one of the most under-recognized causes of gastrointestinal bleeding [2]. A large majority of patients presenting with Dieulafoy’s lesions present with a sudden-onset, massive, recurrent bout of painless hematemesis; however, melena and hematochezia may also be present [5]. Patients may also present with symptoms of anemia, for example, lightheadedness, dizziness, or shortness of breath.

Endoscopy remains the main diagnostic tool for Dieulafoy’s lesions; however, this modality continues to have limitations including, but not limited to, the presence of normal surrounding mucosa, anatomically small in size, and intermittent hemorrhage [5-7]. These limitations are noted after the initial diagnosis [7]. A literature review has demonstrated a high risk of recurrent bleeding with endoscopic monotherapy of 9% to 40%. EGD treatment can be broken down into three groups: regional injection of a sclerosis agent, use of thermal coagulation, and mechanical hemostasis using banding or hemostatic clips [7]. A systematic review has shown mechanical hemostasis using banding and hemoclips is the safest and most effective endoscopic technique used to stop the bleeding of these lesions with a success rate of 95% [2].

Cui and colleagues analyzed the use of three endoscopic hemostasis methods, including Aethoxysklerol injection (46 cases), endoscopic hemoclip hemostasis (31 cases), and a combination of hemoclip hemostasis with Aethoxysklerol injection (30 cases). The purpose of this injection is to act as a sclerosis agent to obstruct the bleeding vessel and further prevent re-bleeding episodes [5]. This study concluded that combined therapy of hemoclip hemostasis with Aethoxysklerol injection was the most effective method for resolving gastrointestinal bleeding in the setting of Dieulafoy’s lesion [5]. This study further emphasizes the importance of endoscopic combination therapy in the management of bleeding from Dieulafoy’s lesions to help mitigate the risk of rebleeding. Therapeutic endoscopy can achieve hemostasis in approximately 90% of accessible lesions, with a <10% risk of rebleeding in the next seven days. Recurrent bleeding can be treated with repeat endoscopic treatment, angiographic embolization, or surgical wedge resection [3]. Our patient had numerous rebleeding episodes resulting in numerous endoscopies that culminated in the embolization of the right gastroepiploic artery. The use of embolization when therapeutic endoscopy has failed, and endovascular management has emerged as first-line therapy for upper GI bleeding refractory to endoscopic management [8].

Three main theories have been presented as possible mechanisms for the development of Dieulafoy’s lesions: age-related mucosal atrophy, wear and tear of the gastric mucosa resulting in the formation of an arterial thrombus with subsequent necrosis and bleeding, and pulsations of the abnormally large artery disrupting the mucosal surface leading to exposure of the artery to bowel contents subsequently resulting in erosion and bleeding [2]. Previous research has noted males are twice as likely to have this lesion compared to females and most commonly in the elderly population, making men in their fifth decade of life the most commonly afflicted by this lesion [2]. Of note, approximately half of patients with diagnosed Dieulafoy's lesions have been noted to be due to NSAIDs, warfarin, and aspirin use [4]. Additionally, diabetes mellitus, chronic kidney disease, cardiovascular disease, and peptic ulcer disease are highly comorbid in patients diagnosed with Dieulafoy's lesions [2]. These lesions typically are located at the lesser curvature of the stomach with bleeding from the left gastric artery [2]. It is important to bring awareness that not all patients fit this description; for example, our patient described in this case.

The rarity of this pathology as a cause of UGIB and multiple, substantial re-bleeding episodes made initial diagnosis and management considerably difficult. Understanding that patients with UGIB may fall outside the typical description of a patient with Dieulafoy's lesion is key in including these lesions as a differential diagnosis and ensuring proper treatment of dual treatment to minimize potential complications such as severe, recurrent gastrointestinal bleeding.

Special considerations

A literature review has shown that the use of NSAIDs, antiplatelets, tobacco, or alcohol may be possible triggers for a potential bleeding event in the setting of Dieulafoy’s lesions [2]. The theory behind the possibility of NSAIDs contributing to bleeding from Dieulafoy’s lesions involves the link between NSAID use and the development of erosive gastritis. Subsequent development of erosive gastritis may lead to necrosis of vessel walls and subsequent rupture of the submucosal vessels [9].

Although this patient denied the use of NSAIDs, she endorsed taking sertraline and Vitamin E. The literature review indicated possible antiplatelet effects from both sertraline and Vitamin E [10-11]. Studies have shown that antiplatelet agents, such as aspirin, clopidogrel, and cilostazol, are associated with the formation of Dieulafoy’s lesions in the upper gastrointestinal tract [11]. While the mechanism behind this association is unknown, the antiplatelet side effects of sertraline and Vitamin E supplementation may contribute to the development of Dieulafoy’s lesions in this patient. Additional studies must be done to further analyze the strength of this association and the mechanism behind how antiplatelets may impact the development of these lesions.

## Conclusions

Early diagnosis and treatment are imperative due to the life-threatening nature of Dieulafoy’s lesions. Patients who do not receive combination endoscopic therapy and only receive endoscopy monotherapy are at a higher risk of re-bleeding. For those that do experience re-bleeding after endoscopic therapy, embolization via angiogram is considered a valid treatment option. Surgical intervention is a last-resort treatment option for those who do not respond to embolization. Further research on all aspects of care for a patient with Dieulafoy’s lesions is necessary to aid in early diagnosis and treatment to better handle this life-threatening condition.
